# A review on the application of the exposome paradigm to unveil the environmental determinants of age-related diseases

**DOI:** 10.1186/s40246-022-00428-6

**Published:** 2022-11-08

**Authors:** Enmin Ding, Yu Wang, Juan Liu, Song Tang, Xiaoming Shi

**Affiliations:** 1 China CDC Key Laboratory of Environment and Population Health, National Institute of Environmental Health, Chinese Center for Disease Control and Prevention, Beijing, 100021 China; 2 Jiangsu Province Center for Disease Prevention and Control, Nanjing, 210009 Jiangsu China; 3 Center for Global Health, School of Public Health, Nanjing Medical University, Nanjing, 211166 Jiangsu China

**Keywords:** Exposomic framework, Aging, Statistical approach, Multiomics, Precise public health

## Abstract

Age-related diseases account for almost half of all diseases among adults worldwide, and their incidence is substantially affected by the exposome, which is the sum of all exogenous and endogenous environmental exposures and the human body’s response to these exposures throughout the entire lifespan. Herein, we perform a comprehensive review of the epidemiological literature to determine the key elements of the exposome that affect the development of age-related diseases and the roles of aging hallmarks in this process. We find that most exposure assessments in previous aging studies have used a reductionist approach, whereby the effect of only a single environmental factor or a specific class of environmental factors on the development of age-related diseases has been examined. As such, there is a lack of a holistic and unbiased understanding of the effect of multiple environmental factors on the development of age-related diseases. To address this, we propose several research strategies based on an exposomic framework that could advance our understanding—in particular, from a mechanistic perspective—of how environmental factors affect the development of age-related diseases. We discuss the statistical methods and other methods that have been used in exposome-wide association studies, with a particular focus on multiomics technologies. We also address future challenges and opportunities in the realm of multidisciplinary approaches and genome–exposome epidemiology. Furthermore, we provide perspectives on precise public health services for vulnerable populations, public communications, the integration of risk exposure information, and the bench-to-bedside translation of research on age-related diseases.

**Supplementary Information:**

The online version contains supplementary material available at 10.1186/s40246-022-00428-6.

## Background

Due to the continuous advancement of biomedical technologies and the substantial improvement in living conditions worldwide, human lifespans have increased dramatically over the past century [1]. Nevertheless, the prevalence of age-related diseases remains considerable: 92 (31.4%) of the 293 diseases listed in the Global Burden of Diseases, Injuries, and Risk Factors Study (GBD) 2017 were identified as age-related, and these accounted for 51.3% of the worldwide burden of disease in adults [2]. In addition, age-related diseases account for two-thirds of human deaths globally and 90% of all deaths in industrialized nations [3]. Moreover, sociodemographic index (SDI) analysis revealed that the rates of age-related diseases ranged from 137.8 disability-adjusted life years (DALYs) per 1000 adults in high-SDI countries to 265.9 DALYs per 1,000 adults in low-SDI countries [2]. The most common age-related diseases are neurological degenerative diseases (e.g., Parkinson’s disease [PD] and Alzheimer’s disease [AD] or dementia), chronic obstructive pulmonary disease (COPD), coronary artery disease (CAD), stroke, type 2 diabetes mellitus (T2DM), and senile deafness [2, 4]. These diseases place an enormous economic and psychological burden on patients, their families, and societies worldwide [5].

In 2021, *Science* published a special issue entitled “125 Questions: Exploration and Discovery.” One of these 125 questions was “Can we stop ourselves from aging?” The U.S. National Institute on Aging (NIA) at the National Institutes of Health (NIH) states that “aging is associated with changes in dynamic biological, physiological, environmental, psychological, behavioral, and social processes.” Although geneticists and epidemiologists have long debated the relative importance of the role played by genotype or the environment in the development of age-related diseases, it is apparent that both can play substantial roles in this process [6, 7]. However, most etiological studies have concentrated on the role of genotype and have considered the environment to play a secondary role. Nevertheless, an analysis of GBD data showed that nearly 50% of deaths worldwide are attributable to environmental exposure, primarily exposure to airborne particulates (including household air pollution and occupational exposure; 14% of all deaths), smoking and secondhand smoke (13%), plasma sodium concentrations (6%), and alcohol consumption (5%) [8]. In contrast, a recent analysis of 28 chronic diseases in identical twins showed that the genetic-related risks of developing one of five age-related diseases were 33.3%, 10.6%, 36.3%, 19.5%, and 33.9% for AD, PD, CAD, COPD, and T2DM, respectively, with a mean of only 26% [9]. The results of over 400 genome-wide association studies (GWASs) have also elucidated that the heritability of degenerative diseases is only approximately 10% [10, 11]. Consequently, nongenetic drivers, such as environmental factors, are now recognized as major risk factors for age-related diseases. The contributions of environmental factors to the development of age-related diseases can be revealed by analyses of all of the factors to which individuals are exposed in their life and the relationships between these exposures and age-related diseases [12, 13].

The concept of the “exposome” was established by C.P. Wild in 2005 as an environmental analogy of the genome and represents the sum of all exposures of an individual throughout the life course [12]. Wild described three categories of the exposome that should be evaluated: (a) specific external exposures, (b) general external exposures, and (c) internal exposures [14]. In this review, we describe the environmental drivers of typical age-related chronic diseases, mainly focusing on AD, PD, CAD, COPD, T2DM, and senile deafness within an exposomic framework. We propose representative strategies for exposomic research on age-related diseases and describe the measurements and statistical approaches that could be used. In addition, we note the challenges of exposomic research and recommend guidelines for the implementation of exposomic concepts in research on age-related diseases.

## Epidemiologic studies on the environmental causes of age-related diseases

### Specific external exposures

#### Air pollution

Atmospheric air pollution is the leading form of specific external exposures, and its role in the development of age-related diseases has been extensively studied. For example, a cohort study in Ontario, Canada, evaluated the association between air pollution and incident PD, which showed that ambient 2.5-µm particulate matter (PM_2.5_) was significantly associated with a 4% increase in incident PD (95% confidence interval [CI] 1.01, 1.08) [15]. Similarly, high concentrations of nitrogen oxides have been found to be significantly associated with an increased risk of AD or dementia in cohort studies in Sweden (hazard ratio [HR]: 1.38; 95% CI 0.87, 2.19) and Taiwan (HR: 1.54; 95% CI 1.34, 1.77) [16, 17]. In the past decade, many studies have explored the relationships between air pollutant exposure and COPD and have determined that the main airborne pollutants affecting the respiratory system are PM_2.5_, 10-µm particulate matter (PM_10_), ozone, carbon monoxide (CO), and sulfur dioxide (SO_2_) [18, 19]. Moreover, a systematic review and meta-analysis showed that short-term exposure to CO (relative risk [RR]: 1.015; 95% CI 1.004, 1.026), SO_2_ (RR: 1.019; 95% CI 1.011, 1.027), nitrogen dioxide (NO_2_) (RR: 1.014; 95% CI 1.009, 1.019), PM_2.5_ (RR: 1.011; 95% CI 1.011, 1.012), and PM_10_ (RR: 1.002; 95% CI 1.000, 1.004) was significantly associated with hospitalization or death due to stroke [20]. Furthermore, multiple cohort studies have shown that exposure to high concentrations of NO_2_ and PM_2.5_ was associated with an increased risk of T2DM [21–25].

#### Chemical contaminants

Some toxic chemicals (e.g., pesticides and heavy metals) have been confirmed to be significantly associated with higher risks of age-related diseases. A meta-analysis of 10 cohort and case–control studies identified that 5 and 10 years of pesticide exposure were associated with a 5% (95% CI 1.02, 1.09) and 11% (95% CI 1.05–1.18) increase in the risk of PD, respectively [26]. A cohort study of 1500 older adult participants aged 65 years or older in southwest France found that occupational exposure to pesticides (e.g., fungicides) was significantly associated with AD in men (RR: 2.29; 95% CI 1.02, 5.63) [27]. Numerous other studies—such as the Agricultural Health Study, the Consortium for the Health Assessment of Great Lakes Sport Fish Consumption, and the Prospective Investigation of the Vasculature in Uppsala Seniors Study—have confirmed that there are associations between exposure to pesticides (especially organochlorine and organophosphate pesticides) and a high risk of T2DM [28–30]. Although it remains unclear whether exposure to heavy metals increases the risk of PD, the data on lead (Pb) exposure suggest that this heavy metal has such an effect. That is, two case–control studies have found a higher risk of PD in the highest Pb exposure quartile than in the lowest Pb exposure quartile (odds ratio [OR]: 2.27 [95% CI 1.13, 4.55] and 3.21 [95% CI 1.17, 8.83]) [31, 32]. Relatively little evidence has been found for an association between heavy metal exposure and AD risk, although there is some evidence that exposure to aluminum (Al) increases the risk of AD. The Personnes Agées QUID (PAQUID) study, a prospective cohort study of almost 4,000 older adults aged 65 years or over in southwest France, found that consumption of Al in drinking water in excess of 0.1 mg per day was associated with a threefold increase in the risk of AD (RR: 3.35; 95% CI 1.49, 7.52) [33]. However, there have been fewer studies of associations of age-related diseases with internal exposures to emerging organic contaminants, such as perfluoroalkyl substances and brominated flame retardants. In one example, the Nurses’ Health Study II determined that higher plasma concentrations of perfluorooctanesulfonic acid (PFOS) or perfluorooctanoic acid (PFOA) were associated with an increased risk of T2DM in women, corresponding to ORs of 1.62 (95% CI 1.09, 2.41) and 1.54 (95% CI 1.04, 2.28), respectively [34]. Similarly, the French E3N prospective cohort study of French women found a possible association between dietary exposure to hexabromocyclododecane (HBCD, HR: 1.47; 95% CI 1.29, 1.67) and polybromodiphenyl ethers (PBDEs, HR: 1.20; 95% CI 1.08, 1.34) and the risk of T2DM [35].

#### Physical factors

Electromagnetic fields and noise are the most common risk factors for age-related diseases, such as neurodegenerative, cardiovascular, and cerebrovascular diseases. A follow-up of 931 participants aged 75 years and older without dementia in Sweden found that long-term occupational exposure to a high electromagnetic field might increase the risk of AD in men (RR: 2.30; 95% CI 1.00, 5.30) [36]. Moreover, short-term exposure to traffic noise was found to be associated with hospitalization due to dementia (RR: 1.15; 95% CI 1.11, 1.20), indicating that noise aggravates the symptoms of dementia [37]. Similarly, a recent meta-analysis and a large Scandinavian population study concluded that road traffic noise increased the incidence of coronary heart disease and stroke by 8% (95% CI 1.01, 1.15) and 6% (95% CI 1.03, 1.08) per 10 A-weighted decibels [dB(A)], respectively [38, 39].

#### Lifestyle and diet

The lifestyle factors most significantly associated with the risk of age-related diseases are smoking, passive smoking, and drinking alcohol. The European Prospective Investigation into Cancer and Nutrition for Parkinson’s Disease, a prospective European population-based cohort study of 220,494 people aged 37 to 70 from 13 centers in 8 countries, found a causal relationship between smoking and PD (HR: 0.70; 95% CI 0.49, 0.99) [40]. A review summarized the existing evidence and confirmed smoking as the main risk factor for COPD [41]. Other environmental exposures, such as dietary exposures and secondhand smoke exposure during pregnancy or early childhood, may also be important risk factors for COPD. A recent cross-sectional analysis of 164,770 adults aged 40 to 69 from the U.K. Biobank found that smoking and passive smoking were associated with a risk of senile deafness (ORs: 1.15 [95% CI 1.09, 1.21] and 1.28 [95% CI 1.21, 1.35], respectively) [42]. Analogously, a recent study of 1,787 mid-to-late-aged adult participants concluded that added salt in the diet might increase at-risk individuals’ risk of AD, while consuming cheese and red wine on a daily basis and lamb on a weekly basis might improve long-term cognitive outcomes [43]. Patel et al. conducted the first exposome-wide association study (EWAS) on T2DM using data from National Health and Nutrition Examination Survey (NHANES) cohorts from 1999 to 2006 [44]. Logistic regression models with multiple comparisons were used, and significant associations for vitamin *γ*-tocopherol (OR: 1.5; 95% CI 1.3, 1.7) and *β*-carotenes (OR: 0.6; 95% CI 0.5, 0.7) were associated with T2DM.

### Internal exposures

#### Medication

The use of antipsychotics, hormones, and anticholinergics has been found to significantly increase the risks of PD and AD. For example, it has been found that among older adults, the use of antipsychotics, such as phenothiazine (RR: 3.65; 95% CI 1.41, 9.45) or benzamide (RR: 2.59; 95% CI 1.23, 5.43), and particularly trichloroethylene (OR: 6.1, 95% CI 1.2, 33), may increase the risk of PD [45, 46]. Moreover, a prospective population-based cohort study in Seattle, Washington (USA), found that higher cumulative anticholinergic use was significantly associated with an increased risk of dementia (HR: 1.54; 95% CI 1.21, 1.96) [47].

#### Gut microbiota

Gut microbial dysbiosis has been found to be associated with many age-related diseases. For example, the fecal samples of PD patients were found to have a substantially lower abundance of *Prevotellaceae* than those of healthy individuals. Additionally, a direct correlation was found between the fecal abundance of *Enterobacteriaceae* and the severity of postural instabilities and gait difficulties [48]. Moreover, the fecal samples of 64 Italian patients with PD exhibited low abundances of gut microbiota (e.g., *Lachnospiraceae*) linked to anti-inflammatory/neuroprotective effects and abnormal concentrations of several classes of fecal metabolites (lipids, vitamins, amino acids, and other organic compounds) [49]. Vogt et al. characterized the bacterial taxonomic composition of fecal samples from participants with and without a diagnosis of dementia due to AD and revealed that the gut microbiome of AD participants had less microbial diversity and was compositionally distinct from that of control individuals. They also identified phylum- to genus-wide differences in bacterial abundance in the microbiome of AD participants, such as a decreased abundance of *Firmicutes*, an increased abundance of *Bacteroidetes*, and a decreased abundance of *Bifidobacterium* [50].

#### Inflammation and oxidative stress

Inflammation is the basis of aging and many age-related chronic diseases, which in turn increase the rate of aging. Walker et al. examined the association between systemic inflammation measured during midlife and 20-year cognitive decline within the Atherosclerosis Risk in Communities cohort study. An increase in the midlife inflammation composite score was associated with an additional 20-year decline of − 0.035 SDs (95% CI − 0.062, − 0.007) in the cognitive composite score [51]. Gong et al. followed three cohort studies in the USA and explored whether proinflammatory diets were associated with increased CVD risks. During 5,291,518 person-years of follow-up, higher dietary inflammatory pattern scores were predefined based on levels of 3 systemic inflammatory biomarkers, which were associated with an increased risk of CVD (HR: 1.38; 95% CI 1.31, 1.46) [52]. Accordingly, age-related diseases can be partly conceptualized as manifestations of accelerated inflammation or aging [53].

#### Hormones

Hormone exposure has been associated with the risk of many age-related diseases, especially among postmenopausal women. Savolainen-Peltonen et al. investigated hormone exposure and AD among Finnish postmenopausal women using Finnish national population and drug register data (1999–2013). The results indicated that long-term use of hormone therapy might be associated with an increased risk of AD (OR: 1.17, 95% CI 1.13, 1.21) [54]. In addition, the pooled results of a systematic review and a time–response meta-analysis showed that there was a significant association between hormone therapy and the risk of AD (OR: 1.08; 95% CI 1.03, 1.14) in menopausal women. However, no comparable association was uncovered for PD [55].

### General external exposures

#### Green space and urbanization

Less adequate evidence has been found for an association between green spaces and cognitive function. In the Cognitive Function and Ageing Studies in England, a higher risk of dementia was found among people who were in the highest quartile of neighborhood natural environment availability than among those in lower quartiles of neighborhood natural environment availability [56]. In contrast, a longitudinal study in the UK demonstrated that a higher level of surrounding greenness in residential areas was associated with a slower cognitive decline among residents over a 10-year follow-up (0.020; 95% CI 0.003–0.037 per interquartile increment) and that this was independent of air pollution exposure [57]. Similarly, a comparison of the effects of the indicators of the built environment on the risk of disease found that urbanization was associated with a higher incidence of COPD (1.05; 95% CI 1.01–1.08 per interquartile increment), while residential green space was associated with a lower incidence of COPD (0.89; 95% CI 0.84–0.93 per interquartile increment) [58].

#### Socioeconomic status and educational level

In a prospective cohort study, social integration was found to contribute to a lower risk of cardiovascular disease (HR: 0.67; 95% CI 0.53, 0.86). In addition, people with the highest social integration level had reduced mortality risks of 30%, 47%, and 53% for AD, COPD, and T2DM, respectively, compared with those with the lowest social integration level [59]. A systematic review and meta-analysis of long-term studies determined that a low social participation index was related to the risk of dementia in terms of social support (RR: 1.28; 95% CI 1.01, 1.62) and social networking (RR: 1.59; 95% CI 1.31, 1.96), while a high social participation index had a moderately protective effect (RR: 0.88; 95% CI 0.80, 0.96) [60]. A cross-sectional study of adults aged 60 or older in China indicated that fewer years of education (OR: 1.55; 95% CI 1.38, 1.73) and being widowed, divorced, or living alone (OR: 2.66; 95% CI 2.29, 3.10) were risk factors for dementia [61]. Another cross-sectional study of older adults with a mean age of 75 years in Denmark showed that compared with individuals with lower household incomes, those with a higher household income were less likely to receive a dementia diagnosis after referral (OR: 0.65; 95% CI 0.55, 0.78) [62]. The search strategies and main results of epidemiologic studies reporting the association between environmental risk factors and age-related diseases (Table S1) in this review are shown in the Additional file 1.

## Potential hallmarks linking the exposome to age-related diseases

Age-related diseases are thought to arise from common underlying processes that precipitate molecular changes over time [63]. These shared mechanisms are grouped into the following hallmarks of aging: genomic instability, epigenetic alterations, telomeric attrition, loss of proteostasis, deregulated nutrient sensing, mitochondrial dysfunction, oxidative stress/damage, inflammation, cellular senescence, stem cell exhaustion, and altered intercellular communication [64, 65]. Some studies have reported the effects of environmental exposures on various hallmarks of aging. For example, exposure to heavy metals and organic pollutants has been associated with mitochondrial dysfunction and DNA methylation alteration in AD [66, 67], and early embryonic exposure to 2,3,7,8-tetrachlorodibenzo-*p*-dioxin may alter global DNA methylation patterns, increasing the risk of T2DM [68]. Bisphenol A concentrations were also found to be associated with increased senescence, inflammation, and decreased telomere lengths in patients with T2DM [69]. However, there have been few other consistent findings confirming that aging hallmarks link the exposome to risks of age-related diseases.

In particular, the alteration of gene expression through DNA methylation, histone modification, and noncoding RNA-associated gene silencing are primary targets of environmental insults considered to induce and sustain epigenetic changes that might result in age-related diseases [70]. In that regard, advances in technology have made it feasible to assay methylation status across the genome in a robust, high-throughput manner, allowing us to address this issue in a data-driven manner. To date, numerous studies have detailed the pattern by which global or site-specific DNA methylation is affected by environmental triggers [67]. For instance, Prado-Bert et al. analyzed the association between the early-life exposome and epigenetic age acceleration based on the Human Early-Life Exposome (HELIX) project. Indoor particulate matter absorbance (PMabs) and parental smoking were found to be positively associated with age acceleration, which was calculated based on Horvath’s skin and blood clock for 1173 children [71]. However, relatively few researchers are looking to understand the functional consequences of methylation marks by assessing gene, protein, and metabolite expression. Recently, Everson et al. identified 443 CpGs that were associated with maternal smoking exposure during pregnancy in seven American, Australian, and European studies. Subsequent expression quantitative trait methylation analyses testing the associations between maternal smoking exposure-associated CpGs and the expression of nearby mRNA showed that exposure-associated CpGs were enriched for environmental response, growth factor signaling, and inflammation [72].

## Limitations of studies examining whether environmental factors contribute to the development of age-related diseases

Although the link between environmental exposure and age-related diseases has been extensively investigated, there are three limitations to previous studies in this area. (1) Most exposure assessments have examined only one chemical or class of chemicals via a traditional targeted measurement approach. However, real-world exposures do not occur in such a manner, and thus, “broad-spectrum” exposure assessments are needed, together with systematic screening of key exposomic elements as well as optimized consideration of the components of exposome domains that affect age-related diseases [73]. (2) Exposure–health association studies have not fully considered the dynamic changes in exposure or the effects of (mixed) exposures in early life stages or in other sensitive time windows on the risks of age-related diseases. (3) Studies have rarely considered chemical absorption, distribution, metabolism, and excretion processes or endogenous biomolecular responses, such as those involving hormones, metabolites, infections, inflammatory reactions, fat peroxidation, oxidative stress, and aging hallmarks.

Therefore, the link between environmental exposure and age-related diseases needs to be explored within an exposomic framework. Such an approach would systematically and substantially increase our understanding of the environmental drivers of age-related diseases, reveal how environmental exposure interacts with genetic susceptibility to age-related diseases, and elucidate the potential biological mechanisms of how these factors affect disease susceptibility, development, and progression.

## Strategies for exposomic research of age-related diseases

Given the limitations of recent research and the evolution of the exposome concept, we propose the following three major strategies for exposomic research of age-related diseases.

1. Comprehensive determination of the chemical compositions of biological fluids and standardized assessment. Pioneers in exposomic research, such as Rappaport, Smith, and Miller, have emphasized the need to fully characterize the chemical composition of biological fluids (e.g., blood and urine) for precisely assessing individuals’ immediate environments and thus their risk of age-related diseases [74, 75]. In addition, standardization of the exposure/outcome assessment methods using conventional tools along with innovative technologies as well as standardized quality assessment tools may contribute to the utility of the exposome concept [73].
2. Consideration of the roles of biological responses and endogenous processes, especially aging biomarkers, at various omics levels (i.e., at the level of the epigenome (e.g., DNA methylation clock), the transcriptome, the proteome, the metabolome, and the microbiome) and with respect to extracellular RNA (including microRNA and long noncoding RNA) abundance and telomere length [76]. Alterations in biomarkers compromise cell and tissue functions and contribute to the incidence of age-related diseases, leading to loss of function and death [77]. In addition, the concentrations of aging biomarkers can reflect the physiological state of individuals and the underlying molecular mechanisms (e.g., genomic instability, epigenetic perturbation, telomeric attrition, loss of proteostasis, altered nutrient sensing, mitochondrial dysfunction, cellular senescence, and disrupted intercellular communications) related to the exposome throughout individuals’ lifespans [64, 78].
3. Performance of exposure and outcome measurements in critical time windows during the aging process. The exposome includes a time dimension, as it emphasizes the importance of assessing exposure throughout life, beginning at the moment of conception. However, a lifelong study of all environmental factors would be highly challenging to perform in real life [79]. Moreover, people age at dissimilar rates, even in terms of tissues and organs, which have a tissue-specific aging signature [80]. In addition, environmental exposure to harmful factors in different critical life windows of susceptibility may lead to distinct disease phenotypes [81, 82]. However, a recent study indicated that there appear to be distinct “waves” of aging over the course of a lifetime at the population level at three ages: 34, 60, and 78 years old [83]. This suggests that exposomic measurements in critical time windows may be useful for the study of the risk of age-related diseases.

**Fig. 1:**
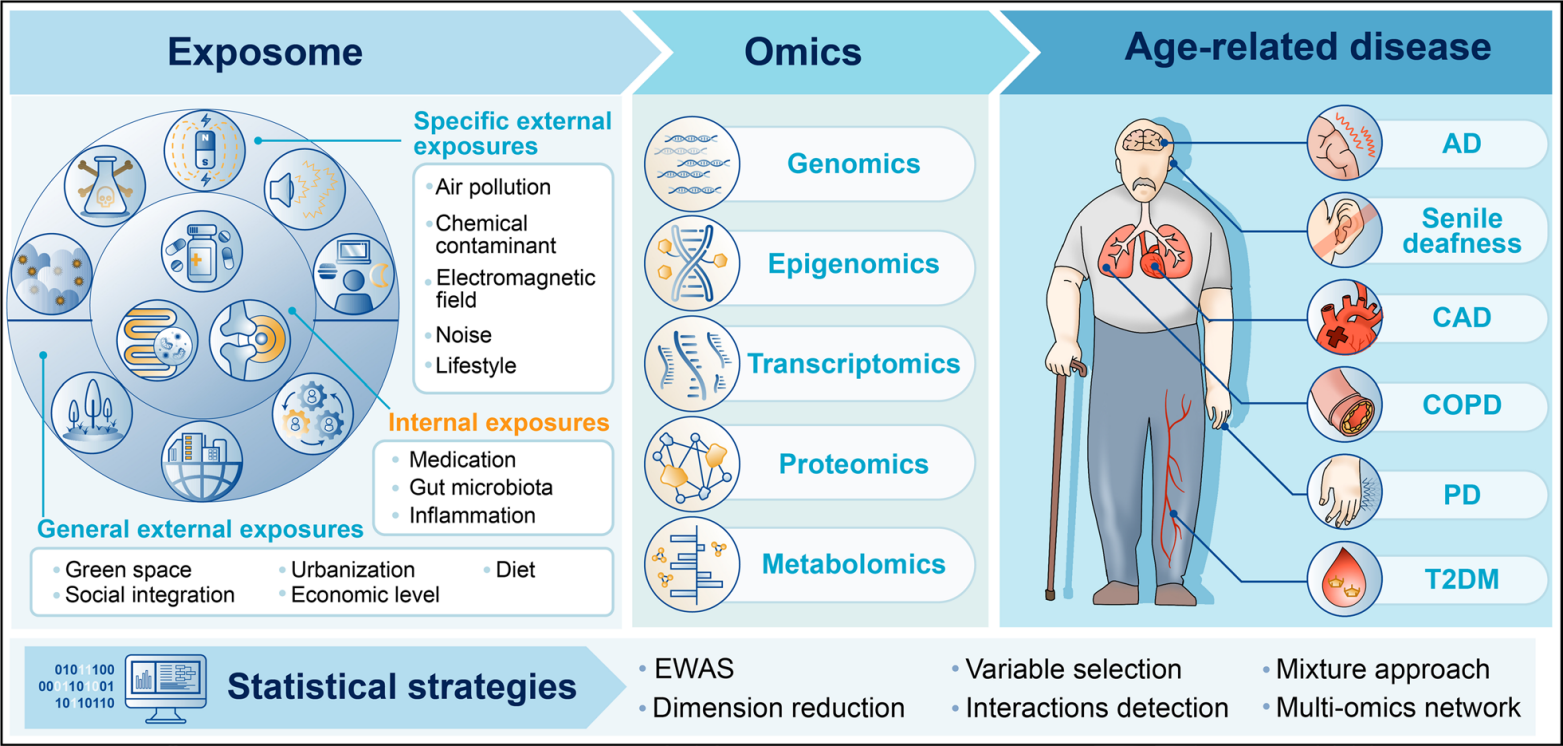
Exposomic analytical framework linking exposures to age-related diseases. The exposome attempts to measure, integrate, and interpret the complex exposures (specific external exposure, general external exposure, and internal exposure) associated with the risk of age-related diseases. The use of multi-omics tools to investigate the adverse effects of these exposures on biological processes can reveal the biological responses to sets of exposures, thereby improving understanding of the development of age-related diseases. Statistical methods can then be applied to identify key elements of the exposome linked to age-related diseases and to determine how these complex exposures affect our biological systems. (AD, Alzheimer’s disease; CAD, cardia-cerebrovascular diseases; COPD, chronic obstructive pulmonary disease; PD, Parkinson’s disease; T2DM, type 2 diabetes mellitus; EWAS, exposome-wide association study)

In summary, systematic approaches are needed to integrate diverse information in exposome research (Fig. 1). The comprehensive evaluation of the relationships between exposures and associated biological responses, such as epigenetic modifications and metabolites, would yield valuable insights into the key elements of the exposome and how environmental factors and their interactions contribute to the risk and development of age-related diseases.

## Exposomic measurement tools for use in age-related disease studies

Exposures can be measured at the population level using geographic information systems (GISs) and remote sensing technologies (such as satellites) or at the individual level using records and surveys, personal sensors, and biological samples. The exposomic information of environmental and biological samples can be measured using advanced analytical techniques, such as high-resolution mass spectrometry (HRMS) and omics platforms.

### Population-level exposures

#### GISs

The use of GISs has changed environmental health research, as GISs integrate databases that connect various attribute data based on geographic location (e.g., residential address). Therefore, GISs can quantify the buffer distance between exposure sources and human receptors and can be used to describe the proximity of roads, factories, green spaces, water bodies, and other features that may result in exposures that negatively (e.g., farms, which may result in exposure to pesticides) [84] or positively (e.g., health food stores or entertainment venues) affect human health [85].

#### Remote sensing

Remote sensing is the science of obtaining information about objects or regions from a distance (usually from airplanes or satellites). Remote sensing technology has been used to estimate the levels of various environmental exposures, such as PM_2.5_ [86], NO_2_ [87], green space [88], temperature [89], building environment [90], outdoor light at night [91], and pesticides [92]. For example, global PM_2.5_ concentrations were estimated from information from three satellites in conjunction with a chemical transport model and ground-based sun photometer observations [93]. Remote sensing estimates have also been used to assess the relationships between PM_2.5_ exposure and cardiovascular diseases [25, 94, 95]. Moreover, recent studies have used a 1-km valuation of U.S. PM_2.5_ concentrations, thereby increasing the utility of such measurements in exposomic studies [96, 97].

### Individual-level exposures

#### Portable/personal sampling and sensing devices

An increasing number of personal sensors are being developed to monitor heart rate, blood sugar concentrations, blood pressure, muscle activity, temperature, and sweat production [98]. Remarkable advances in novel algorithms in wearable technologies are allowing for accurate acquisition of personal temperature exposures, chemical and biological exposures, and clinical laboratory measurements to explore individual-level 24-h exposures in various microenvironments in exposome studies [99–102]. Koelmel et al. captured the changes in gas- and particulate-phase airborne chemicals to which individuals were exposed and determined which exposures posed the greatest risk to health. They used wearable exposure monitors, i.e., passive air-sampling wristbands, which were provided to 84 healthy participants (aged 60–69) as part of the Biomarkers for Air Pollution Exposure (China BAPE) study [103, 104]. Jiang et al. developed a sensitive method to monitor personal airborne biological and chemical exposures, which enabled them to follow the personal exposomes of 15 individuals for up to 890 days over 66 distinct geographical locations. They found that humans are exposed to thousands of microbial species with great intraspecies diversity and demonstrated that the human exposome is highly dynamic and influenced by spatial/lifestyle and seasonal variables [105, 106].

#### Records and surveys

Data from health records and surveys can be used to describe the exposure characteristics of individuals at a relatively low cost. For example, electronic health records provide an efficient way to estimate individual-level exposure to, for example, pharmaceutical drugs, alcohol, or cigarette smoke. Similarly, surveys performed as part of epidemiological studies can provide valuable information on peoples’ diet, socioeconomic status, education level, and psychosocial stress level.

#### Biological samples

The analysis of biological samples (e.g., blood, urine, saliva, feces, and hair) enables quantification of the burden of chemical exposures for an individual. In particular, abundant information on the biological response to an exposure can be obtained by quantifying small molecules in biological samples, such as inflammatory factors and metabolites.

### Analytical platforms

#### HRMS

Advancements in HRMS have provided new tools to identify and quantify multiple chemical substances in a wide range of media and have extended the analytical window beyond the targeted profiling of known metabolites and priority pollutants [107]. In particular, HRMS enables the simultaneous analysis of many exogenous and endogenous compounds and the characterization of the state of a biological system and its response to environmental factors [13, 108].

Nontargeted HRMS-based analysis can collect data on thousands of chemical features in a single analytical run and is thus suitable for mass screening, although most of these features remain unannotated [109]. However, targeted HRMS-based analysis involves performing multiple analyses of known and specific chemicals, including absolute qualitative and quantitative analyses of the substances to be measured using certified standards, and is therefore suitable for validation in exposomic studies. For example, as part of the China BAPE study [104], Guo et al. performed targeted HRMS-based analysis of 70 airborne compound exposures of 84 elderly people in Jinan, China, and thereby described their personal exposure to air pollutants in terms of concentration and distribution [110].

#### Multiomics technology

The broad diversity of omics biomarkers that have been used to assess biological responses provides new opportunities to understand the impact of the environment on the risk of age-related diseases. For example, the multiomics analysis and integration method produces a priority list of multiple sets of biomarkers, which together reflect the molecular responses of the exposome. Each of these data warrants integration into a biomarker panel to aid physicians in developing age-related disease diagnoses and prognoses [78].

By integrating the response measures of genomics, epigenomics, transcriptomics, proteomics, and metabolomics, it is possible to develop a systems biology-level understanding of how exposure affects key biochemical processes. Similarly, the aggregated biological response model, which combines toxicology and pharmacology with molecular and environmental epidemiology, represents a new paradigm for delineating the mechanisms of chemical toxicology [13]. The further development of statistical methods to determine the interactions between biological response networks [111, 112] and the application of multiomics methods in cohort studies to elucidate the characteristics of human exposure [113, 114] will facilitate exposomic discoveries in the years ahead. For instance, the EXPOsOMICS project, an integration of 14 different studies, applied multiomics technologies such as metabolomics and adductomics to quantify exposure levels and to measure downstream effects of environmental exposures by the epigenome, transcriptome, and proteome [114]. The PISCINA-II study (part of the EXPOsOMICS project) included 60 volunteers who swam for 40 min in a chlorinated pool, and metabolome-wide association showed that disinfectant byproducts were associated with 333 metabolic features, of which 13 metabolites were identified and enriched in the tryptophan metabolism pathway [115].

Recently, more research has incorporated multiomics technologies and personal sampling and sensing devices to reveal the dynamic molecular effects of precise personal exposure. For example, Jiang et al. developed a personal wearable device that monitored airborne biological and chemical exposures and followed the personal exposomes of 15 individuals for nearly 3 years [105]. Next-generation sequencing and liquid chromatography‒mass spectrometry (LC‒MS) technologies were then used to detect the biotic/chemical exposome and found that the human exposome was dynamic and influenced by various environmental and spatial/lifestyle variables. Gao et al. examined the effects of environmental exposures on well-dissected personal internal health changes based on longitudinal individual exposomes and internal multiomics (gut microbiome, proteome and metabolome) [101]. By investigating the correlations of exposomics and the exposome and clinical data, it was found that changes in external exposures could be associated with thousands of biomolecular changes in the body involved in immune, kidney, and liver functions.

## Evolving statistical methods in exposome–health association studies of age-related diseases

### Exposome-wide association study (EWAS)

An EWAS is a statistical method similar to a GWAS that is used to continuously and independently detect the association of many exposures with a set of results (with adjustment for potential confounding factors). Although nonideal, for practical purposes—because exposure studies examine many exposures—the same set of confounding factors is typically used for all exposures. Furthermore, multiple comparison corrections should be applied to minimize the number of false-positive results obtained. Bonferroni–Holm correction method (family-wise error rate [FWER] method) or the Benjamini–Hochberg correction method (false discovery rate [FDR] method) are among the most commonly used multiple comparison correction methods in epidemiological research. However, a limitation is that these methods assume that tests are independent, and if this assumption is violated, these methods may generate highly conservative results [116]. The Bonferroni correction method has been devised and involves dividing the significance level *α* by the test number *M* (*α*_corrected_ = *α*/*M*); this changes the value of *M* according to the number of valid checks, as determined by the relevant structure of the data [116, 117]. Methods such as stepwise multiple testing have been developed to capture the joint dependency structure of the test statistic, thereby improving the ability to detect false hypotheses [118]. However, there has been no development of multiple-detection correction methods to simultaneously deal with the diversity of exposure and the results of exposure environments. In a study that simulated the true correlation structure of the Infancia y Medio Ambiente (INMA, Spain) birth cohort exposure variables, EWAS exhibited a higher FDR than other methods but showed the greatest sensitivity [119].

### Variable selection tools

The most popular variable selection techniques in association research include the deletion/substitution/addition (DSA) algorithm, the elastic net (ENET) algorithm, and the graphical unit evolutionary stochastic search (GUESS) algorithm. For example, the DSA algorithm was used in the recent HELIX project to explore the relationship between early-life exposure to environmental hazards and childhood lung function and the relationship between urban pregnancy exposure to environmental hazards and birth weight [120, 121]. In simulation research, the DSA method has shown higher sensitivity and a lower FDR than other methods and good performance in capturing interactive items [119, 122]. However, the DSA method has routinely been criticized because its estimations are inconsistent if the ratio of the sample size to the number of candidate predictions is small, and its confidence intervals are low if there is a large correlation between predictions [123].

The ENET algorithm has been used to study the relationship between various environmental pollutants and birth weight in three cohorts, one each in Greenland, Poland, and Ukraine, and to examine the relationship between concentrations of several persistent organic pollutants in breast milk samples and infant behavioral problems [124, 125]. In addition, the ENET method showed high sensitivity and a moderate FDR in a simulation scenario [119].

The GUESS algorithm is a Bayesian variable selection technique and is a search algorithm based on a multichain genetic algorithm. It was developed to explore complex genetic association models and maximize the detection of genetic variation [126]. Similar to the DSA algorithm, the GUESS algorithm showed high sensitivity and a low FDR in simulation research [119]. However, to explain confounding factors, the results must first be fitted to these confounding factors, and the GUESS model then fitted to the residuals.

### Dimension reduction techniques

Dimensional reduction techniques such as factor analysis or principal component analysis (PCA) have been used to analyze the impact of multiple pollutants on the risk of age-related diseases [127–129]. PCA uses the eigenvalues and eigenvectors of the exposure correlation matrix, makes full use of data variability, and derives orthogonal components from the set of exposure variables. The resulting “feature exposure” can be used for subsequent analysis. However, the relationship PCA determines between exposure and response variables does not take into account the generation of principal components. An improved version of PCA, supervised principal component analysis, can solve this problem [130], as it eliminates the prescreening results of pollutants that are not related to the result and thereby generates an estimated effect with a smaller deviation than the corresponding PCA estimate. The PISCINA-II study (part of the EXPOsOMICS project) aimed to apply the partial least squares (PLS) regression method to investigate the effect of disinfectant product exposure on inflammation [115, 131]. Sparse PLS (sPLS) regression has previously been used in research, such as to identify multiple pollutant exposure profiles related to male reproductive function biomarkers [132]. This method constructs latent variables (linear combinations of predictors) in a supervised manner, that is, uses the results and then regresses the results on the latent variables. The sPLS component not only captures as much predictor variance as possible but also focuses on the variance associated with the results of interest. In a simulation setting, this method showed high sensitivity and a moderate FDR [119].

### Interaction detection models

In a recent paper, Barrera-Gómez considered scenarios with statistical interactions and systematically compared methods recommended for search interactions [119]. The simulation used contained 237 exposures with real structures, and several statistical regression methods were used. These methods were the two-step full environment association study, the DSA algorithm, the least absolute shrinkage and selection operator (LASSO), and the group-LASSO interaction network (GLINTERNET), which is a three-step method based on a regression tree and an enhanced regression tree. The GLINTERNET and DSA methods performed equally well in terms of the ability to capture interactive items but exhibited an identical trade-off between sensitivity and FDR.

The Bayesian model average (BMA) method is another statistical method used for the construction of a health risk model that considers multiple pollutants and their interactions. The BMA method is effective for dealing with model uncertainty, as it provides robust estimates of parameters through model averaging. That is, compared with traditional modeling methods that ignore model uncertainties, the BMA method is attractive because it does not choose a single “best” model but instead makes average inferences within a range of possible models [133, 134]. In a simulation study by Sun et al., the BMA method proved useful for the selection of variables with moderately strong exposure–response associations [135].

### Mixture approaches

Notably, most of the current chemical exposure–health effect analyses are based on the risk assessment of individual compounds rather than chemical mixtures. However, even exposure to a single pollutant or dose might not cause detrimental health effects, the simultaneous and cumulative exposure to a variety of pollutants may induce the adverse health outcomes. For mixture effect analysis, numerous statistical approaches, such as weighted quantile sum (WQS) regression, Bayesian kernel machine regression (BKMR), and quantile G-computation, have been developed to interpret the overall effect of chemical coexposure on health outcomes and the relative importance of each exposure [136–138]. Recently, Caporale et al. used data from the Swedish Environmental Longitudinal, Mother and child, Asthma and allergy (SELMA) pregnancy cohort and developed a mixture-centric risk assessment strategy integrating epidemiological and experimental data [139]. By applying WQS regression to establish associations between endocrine-disrupting chemical (EDC) mixture exposure and language delay in children, they found that exposure to EDC mixtures in early pregnancy was associated with language delay in offspring. This study highlights the need to consider mixtures in chemical testing and risk assessment processes and provides a comprehensive framework to guide risk assessment strategies.

Overall, the above studies have shown that systems approaches are needed to integrate diverse information in exposomic research (Fig. 1). The systematic evaluation of the relationship between exposures and associated biological responses, such as epigenetic modifications and metabolites, provides valuable insights into key elements of the exposome and delineates how environmental factors and their interactions contribute to the risk of age-related diseases. The statistical strategy in this framework usually contains (I) an EWAS model to explore the associations of exposure–health outcomes; (II) variable selection tools as well as dimension reduction techniques for exposure screening and feature dimension reduction; (III) interaction detection models as well as mixture approaches for assessing the mixture effect, relative contribution, and interactions of chemical coexposure; and (IV) a multiomics network linking exposure to disease phenotype by elucidating their underlying biological mechanisms.

## Challenges and opportunities

### Multidisciplinary approaches

Each of the subdisciplines of environmental health science can aid in the elucidation of the connection between the exposome and age-related diseases. Information and findings from geriatric populations (via geriatric epidemiology), accurate measurements of chemical species in the environment (via exposure science), exposome–health outcome association analysis (via biostatistics), and data on biological mechanisms and target pathways (via toxicology, molecular biology, and bioinformatics) will all be needed for exposomic research. The findings from these major subdisciplines will need to be integrated via computational methods, together with data from analytical chemistry, genomics, behavioral science, nutritional sciences, and many other fields.

Environmental health science is most powerful when it capitalizes on the transfer of knowledge between its three major subdisciplines: toxicology, geriatric epidemiology, and exposure science. Toxicologists rely on geriatric epidemiological studies and the analysis of exposures to determine which compounds linked to age-related diseases should be studied. In turn, geriatric epidemiologists rely on toxicologists to determine if their observed associations are in line with what is known about the biological and toxicological pathways involved in the aging process or age-related diseases. Toxicologists also rely on exposure scientists to determine what levels of exposure are relevant. If data from all three subdisciplines agree that a particular environmental exposure has an impact on the risk of age-related diseases, this is very strong scientific evidence that such an impact is real. A similar level of convergence among all components of environmental health sciences will be necessary for data from the exposome to have an impact on studies of the risks of age-related diseases.

### Genome–exposome epidemiology

The gene–environment (G × E) interaction is a key driver of human health and age-related diseases. The genetic coding system that has evolved to allow individuals to adapt to the environment is supported by a memory system to improve survival and reproduction [140, 141]. Ultimately, genetic epidemiology and exposure epidemiology must fully incorporate this interaction to account for the complex accumulation of G × E interactions over an individual’s lifetime. Given that the exposome is the accumulation of environmental impacts and related biological responses throughout the entire lifespan [76], predictive models of health outcomes and age-related diseases must comprehensively understand the genetic memory system, as it enables individual genomes to learn from their exposure history and thereby improve their response to subsequent exposures.

Exposure epidemiology is a necessary condition to solve the G × E (∫G × E) integral, which is a function of E and is thus constantly changing throughout an individual’s life. However, when the number of interacting elements exceeds 10^18^ (20,000 genes × 20,000 splicing variant transcripts × 100,000 or more proteins × 20 or more posttranslational modifications × 10,000 or more metabolites), it is impractical to conduct a systematic investigation of all assumptions [142]. One solution would be to obtain an informative longitudinal analysis of personal genetic and exposure information, as although the initial power would be limited, this would provide a platform to achieve long-term goals and clarify the level of the population whose exposure affects the results [143].

When discussing the analytical complexity inherent to determining an underlying G × E interaction, it should be noted that a statistical interaction does not represent a biological interaction [144]. However, by using emerging and abundant biological data sources, other potential signals can be identified from the large space of potential interactions [145]. Recently, sophisticated methods have been developed for exploring large-scale G × E interactions, and these methods could help solve the problem of dealing with many variables [9, 146].

At present, a few studies have examined interactions between sequence variations in the whole genome and environmental exposure to identify novel genetic modulators for certain environmental factors in age-related diseases. For instance, Biernacka et al. performed the first genome-wide gene‒environment interaction analysis of pesticide exposure and risk of PD using data on > 700,000 single nucleotide polymorphisms (SNPs) in 364 discordant sibling pairs [147]. They applied a PCA and logistic regression model to detect SNP–pesticide interactions and found that the effect of pesticide exposure on PD risk may be modified by SNPs of the *ERCC6L2* gene. Patel et al. used the NHANES and screened 18 SNPs and 5 serum-based environmental factors (results from previous GWASs and EWASs) for interactions in association with T2DM [148]. The results showed that the interaction between rs13266634 (*SLC30A8*) and trans-*β*-carotene withstood Bonferroni correction (*P* = 0.006), and the per risk allele effect sizes among subjects with low levels of trans-*β*-carotene were 40% greater than the marginal effect size (OR: 1.8; 95% CI 1.3–2.6). Nonetheless, understanding the complex interplay between genes and the environment based on whole genome-wide and whole exposome-wide designs and more sophisticated models that incorporate the complexity of exposure mixtures, multiple variants, and multiple types of genomic data (epigenetic, genetic, transcriptomic, etc.) will likely shed new insights on this issue.

## Perspectives and conclusions

The development of the exposome concept and related technologies will have several impacts on precise public health research and delivery.

1. The first impact will concern vulnerable populations, as the exposome offers an overall view and integrates the source of such populations’ vulnerability with the other exposures that an individual may have and which can aggravate or cause a pathological condition. These vulnerabilities can relate to life stage (e.g., affect children or older adults), sex, genetic factors, dietary origin, pathologies, or particular socioeconomic conditions [149, 150]. For example, older adults are generally considered a susceptible population because of the gradual decline in physiological processes over time. In addition, dosimetric studies showed that there was reduced clearance of PM in all regions of the respiratory tract with increasing age beyond young adulthood [151].
2. The second impact will concern the communication and dissemination of public health messages. Such messages regarding age-related diseases can be contradictory, e.g., “eat more vegetables” but “avoid exposure to pesticides.” Thus, a more integrated approach would give more credibility to these messages.
3. The third impact will concern perspective data reflecting exposures, which can be incorporated into precision medicine to complement genetic information. At a large scale, a systems biology approach can integrate various levels of heterogeneous information. This could include both genetic data and exposomic data, including multiomics data, thereby facilitating a more comprehensive analysis of disease etiologies and specific vulnerabilities than has been provided by previous studies. The subsequent challenge will be to more broadly integrate various risk exposures to obtain a global and more realistic overview of exposure–genetic interactions [152–154].
4. The last impact will involve the bench-to-bedside translation of research. The increasing combination of exposomic data with multiomics data will enable researchers to readily identify interim biomarkers of exposure and response that will reveal subtle biological changes. These will facilitate early diagnosis and the identification of potential intervention targets for the prevention and treatment of age-related diseases. In addition, primary prevention to reduce key exposures would reduce the contribution of environmental stressors to the development of age-related diseases and minimize the adverse effects of chemical stressors on individuals as they age.

## Conclusions

The explosion in exposome research related to aging and the risk of age-related diseases may provide an answer—from an environmental perspective—to the question “Can we stop ourselves from aging?”, as recently posed by *Science* magazine.

## Supplementary Information

**Additional file 1.** The search strategies and main results of epidemiologic studies reporting the association between environmentalrisk factors and age-related diseases.

## Data Availability

Data sharing is not applicable to this article as no datasets were generated or analyzed during the current study.

## Abbreviations

GBD: Global Burden of Diseases, Injuries, and Risk Factors Study
SDI: Socio-demographic index
DALYs: Disability-adjusted life years
PD: Parkinson’s disease
AD: Alzheimer’s disease
COPD: Chronic obstructive pulmonary disease
CAD: Coronary artery disease
T2DM: Type 2 diabetes mellitus
GWAS: Genome-wide association study
HR: Hazard ratio
OR: Odds ratio
RR: Relative risk
CI: Confidence interval
PM_2.5_: 2.5-µM particulate matter
PM_10_: 10-µM particulate matter
CO: Carbon monoxide
SO_2_: Sulfur dioxide
NO_2_: Nitrogen dioxide
Pb: Lead
Al: Aluminum
PFAS: Per- and polyfluoroalkyl substances
dB(A): A-weighted decibels
GIS: Geographic information system
HRMS: High-resolution mass spectrometry
EWAS: Exposome-wide association study
FDR: False discovery rate
DSA: Deletion/substitution/addition
ENET: Elastic net
GUESS: Graphical unit evolutionary stochastic search
PCA: Principal component analysis
PLS: Partial least squares
sPLS: Sparse PLS
LASSO: Least absolute shrinkage and selection operator
GLINTERNET: Group-LASSO interaction network
BMA: Bayesian model average
G × E: Gene–environment interaction
