# Supplementary material for: A review on the application of the exposome paradigm to unveil the environmental determinants of age-related diseases
Source: Hum Genomics. 2022 Nov 8;16:54. doi: 10.1186/s40246-022-00428-6 (PMC9644545; doi:10.1186/s40246-022-00428-6)
Supplement: Supplementary file 1 — Additional file 1. The search strategies and main results of epidemiologic studies reporting the association between environmentalrisk factors and age-related diseases. [file 40246_2022_428_MOESM1_ESM.docx]

**Supporting Methods**

**Search strategy for current epidemiologic studies on the environmental causes of age-related diseases**

We searched PubMed and Embase database for epidemiological studies reporting an association between certain environmental exposure and age-related diseases. The following inclusion criteria was applied to identity eligible studies: (1) the study design included prospective cohort study, retrospective cohort study, case-control study, cross-sectional study, and systematic review/meta-analysis; (2) the studies include relatively clear disease outcomes; and (3) the studies were conducted in elderly population aged 60 years and older.

**Table S1. Epidemiologic studies reporting the association between environmental factors and age-related diseases**

| **Category** | **Exposure** | **Disease** | **Study design** | **Methodology** | **Major finding** | **Effect estimate** | **Association** |
| --- | --- | --- | --- | --- | --- | --- | --- |
| Air pollution | PM_2.5_ | PD | Retrospective cohort | Cox proportional hazards models | PM_2.5_ was significantly associated with a 4% increase in incident PD | HR: 1.04; 95%CI: 1.01, 1.08 | Positive[1] |
| Air pollution | NO | AD | Prospective cohort | Cox proportional hazards models | Nitrogen oxide levels have been found to be significantly associated with an increased risk of AD | HR: 1.38; 95%CI: 0.87, 2.19 | Positive[2] |
| Air pollution | NO_2_ | AD | Retrospective cohort | Cox proportional hazards models | High concentrations of nitrogen oxides have been found to be significantly associated with an increased risk of AD or dementia | HR: 1.54; 95%CI: 1.34, 1.77 | Positive[3] |
| Air pollution | PM_2.5_ | COPD | Retrospective cohort | Cox proportional hazards models | Long-term exposure to fine particulate matter associated with development of COPD in the elderly | HR: 1.09; 95%CI: 1.07, 1.11 | Positive[4] |
| Air pollution | O_3_; CO; NO_2_; SO_2_; PM_10_; PM_2.5_ | COPD | Meta-analysis | Meta | Short-term exposure to air pollutants increases the burden of risk of COPD acute exacerbations significantly | SO_2_ (RR: 1.012; 95%CI: 1.001, 1.023), NO_2_ (RR: 1.019; 95%CI: 1.014, 1.024) | Positive[5] |
| Air pollution | PM_2.5_; PM_10_; CO; SO_2_; NO_2_; O_3_ | CAD | Meta-analysis | Meta | Short-term exposure to CO, SO_2_, NO_2_, PM_2.5_ and PM_10_ is significantly associated with hospitalization or death due to stroke | CO (RR: 1.015; 95%CI: 1.004, 1.026), SO_2_ (RR: 1.019; 95%CI: 1.011, 1.027), NO_2_ (RR: 1.014; 95%CI: 1.009, 1.019), PM_2.5_ (RR: 1.011; 95%CI: 1.011, 1.012), PM_10_ (RR: 1.002; 95%CI: 1.000, 1.004) | Positive[6] |
| Air pollution | NOx; PM_2.5_ | T2DM | Prospective cohort | Cox proportional hazards models | Exposure to traffic-related pollutants may increase the risk of T2DM | PM_2.5_ (HR: 1.63; 95%CI: 0.78, 3.44), NOx (HR: 1.25: 95%CI: 1.07, 1.46) | Positive[7] |
| Air pollution | NO_2_; NOx | T2DM | Prospective cohort | Cox proportional hazards models | Long-term exposure to traffic-related air pollution may contribute to the development of diabetes | HR: 1.04; 95%CI: 1.00, 1.08 | Positive[8] |
| Air pollution | PM_2.5_; NO_2_ | T2DM | Prospective cohort | Cox proportional hazards models | Fine particulate matter may relevant pollutant for diabetes development | PM_2.5_ (HR: 1.11; 95%CI: 1.02-1.22), NO_2_ (HR: 1.05; 95%CI: 0.99-1.12) | Positive[9] |
| Air pollution | PM_2.5_ | T2DM | Prospective cohort | Cox proportional hazards models | Long-term exposure to PM_2.5_ may contribute to the development of diabetes | HR: 1.11; 95%CI: 1.02, 1.21 | Positive[10] |
| Chemical contaminants | Pesticide | AD | Meta-analysis | Meta | pesticide exposure was associated with an increase in the risk of PD | OR:1.11; 95%CI: 1.05, 1.18 | Positive[11] |
| Chemical contaminants | Pesticide | AD | Prospective cohort | Cox proportional hazards models | Occupational exposure to pesticides was significantly associated with AD | RR: 2.29; 95%CI: 1.02, 5.63 | Positive[12] |
| Chemical contaminants | Pesticide | T2DM | Prospective cohort | Logistic regression | Applicators who had used the organochlorine insecticides aldrin, chlordane, and heptachlor more than 100 lifetime days had increased odds of diabetes | Aldrin:1.51, chlordane:1.63, heptachlor:1. 94 | Positive[13] |
| Chemical contaminants | Pesticide | T2DM | Prospective cohort | Cox proportional hazards models | DDE exposure was associated with incident diabetes | RR: 2.37; 95%CI: 1.20, 4.66 | Positive[14] |
| Chemical contaminants | Pesticide | T2DM | Prospective cohort | Logistic regression | Environmental exposure to some POPs substantially increased risk of future T2DM in an elderly population | OR: 3.4; 95%CI: 1.0, 11.7 | Positive[15] |
| Chemical contaminants | Lead | PD | Case-control | Logistic regression | Occupational exposure to Pb is a risk factor for PD | OR: 2.27; 95%CI: 1.13, 4.55 | Positive[16] |
| Chemical contaminants | Lead | PD | Case-control | Logistic regression | Cumulative exposure to lead increases the risk of PD | OR: 3.21; 95%CI: 1.17, 8.83 | Positive[17] |
| Chemical contaminants | Aluminum | AD | Prospective cohort | Cox proportional hazards models | Consumption of Al in drinking water in excess of 0.1 mg per day was associated with a threefold increase in the risk of AD | RR: 3.35; 95%CI: 1.49, 7.52 | Positive[18] |
| Chemical contaminants | PFAS | T2DM | Prospective nested case-control | Logistic regression | Higher plasma concentrations of perfluorooctanesulfonic acid or perfluorooctanoic acid were associated with an increased risk of T2DM | PFOS (OR: 1.62: 95%CI: 1.09, 2.41), PFOA (OR: 1.54; 95%CI: 1.04, 2.28) | Positive[19] |
| Chemical contaminants | Brominated flame retardants | T2DM | Prospective cohort | Cox proportional hazards models | A possible association between dietary exposure to hexabromocyclododecane and polybromodiphenylethers and the risk of T2DM | HR: 1.47; 95%CI: 1.29, 1.67 | Positive[20] |
| Physical factors | High electromagnetic field | AD | Prospective cohort | Cox proportional hazards models | Long-term occupational exposure to a high electromagnetic field may increase the risk of AD | RR: 2.30; 95%CI: 1.00, 5.30 | Positive[21] |
| Physical factors | Traffic noise | Dementia | Longitudinal time-series | Generalized linear model | Short-term exposure to traffic noise was found to be associated with hospitalization due to dementia, indicating that noise aggravates the symptoms of dementia | RR: 1.15; 95%CI: 1.11, 1.20 | Positive[22] |
| Physical factors | Traffic noise | CAD | Cohort study | Cox proportional hazards models | Road-traffic noise increased the incidence of coronary heart disease and stroke | HR: 1.06; 95%CI: 1.03, 1.08 | Positive[23] |
| Lifestyle and diets | Smoking | PD | Prospective cohort | Cox proportional hazards models | A causal relationship between smoking and PD | HR: 0.70; 95%CI: 0.49, 0.99 | Negative[24] |
| Lifestyle and diets | Smoking | Deafness | Cross-sectional | Logistic regression | Smoking and passive smoking were associated with a risk of senile deafness | Current smoke (OR: 1.15, 95%CI: 1.09, 1.21), Passive smoke (OR: 1.28; 95%CI: 1.21, 1.35) | Positive[25] |
| Lifestyle and diets | Diets | AD | Prospective cohort | Structural equation modeling | Added salt in the diet may increase at-risk individuals’ risk of AD | Not applicable | Negative[26] |
| Lifestyle and diets | γ-tocopherol; β-carotene | T2DM | Retrospective cohort | Logistic regression | Significant associations for the γ-tocopherol and β-carotene were discovered to be associated with T2DM | γ-tocopherol (OR: 1.5; 95%CI: 1.3, 1.7), β-carotene (OR: 0.6; 95%CI: 0.5, 0.7) | γ-tocopherol: Positive, Beta-carotenes: Negative[27] |
| Medication | Neuroleptics | PD | Cohort | Cox proportional hazards models | The risk of probable PD was increased by 3.2-fold after exposure to neuroleptics | Benzamides (RR: 3.65; 95%CI: 1.41, 9.45), Phenothiazines (RR: 2.59; 95%CI: 1.23, 5.43) | Positive[28] |
| Medication | Trichloroethylene | PD | Cohort | Logistic regression | Ever exposure to trichloroethylene was associated with significantly increased risk of PD | OR: 6.1, 95%CI: 1.2, 33 | Positive[29] |
| Medication | Anticholinergic | Dementia | Prospective cohort | Cox proportional hazards models | Higher cumulative anticholinergic use was significantly associated with an increased risk of dementia | HR: 1.54; 95%CI: 1.21, 1.96 | Positive[30] |
| Gut microbiota | Gut microbiota | PD | Case-control | Logistic regression | The fecal samples of PD patients were found to have a significantly lower abundance of Prevotellaceae than those of healthy individuals | Not applicable | Negative[31] |
| Gut microbiota | Gut microbiota | PD | Case-control | Generalized linear model | Low abundances of gut microbiota linked to anti-inflammatory/neuroprotective effects, and abnormal concentrations of several classes of fecal metabolites | Not applicable | Negative[32] |
| Gut microbiota | Gut microbiota | AD | Case-control | T-test | The gut microbiome of AD participants had less microbial diversity and was compositionally distinct from that of control individuals | Not applicable | Negative[33] |
| Inflammation and oxidative stress | Inflammation | Cognitive impairment | Prospective cohort | Generalized estimating equations | Increase in midlife inflammation composite score was associated with an additional 20-year decline of -0.035 SD on the cognitive composite score | -0.035; 95%CI: -0.062, -0.007 | Negative[34] |
| Inflammation and oxidative stress | Inflammation | CVD | Prospective cohort | Cox proportional hazards models | Dietary patterns with a higher proinflammatory potential were associated with higher CVD risk | HR: 1.38; 95%CI: 1.31, 1.46 | Positive[35] |
| Hormones | Hormones | AD | Case-control | Logistic regression | Long term use of systemic hormone therapy might be accompanied with an overall increased risk of Alzheimer's disease | OR: 1.17, 95% CI: 1.13, 1.21 | Positive[36] |
| Hormones | Hormones | AD | Meta-analysis | Meta | There was a significant association between hormone therapy and the risk of AD | OR: 1.08; 95%CI: 1.03, 1.14 | Positive[37] |
| Green space and urbanization | Community environment | Cognitive impairment | Prospective cohort | Logistic regression | Increased odds of dementia and cognitive impairment were found in the highest quartile of natural environment availability | Dementia (OR: 2.2; 95%CI: 1.2, 4.2), Cognitive impairment (OR: 1.4; 95%CI: 1.0, 2.0) | Negative[38] |
| Green space and urbanization | Green space | Cognitive impairment | Prospective cohort | Linear mixed effects models | A higher level of surrounding greenness in residential areas was associated with a slower cognitive decline in residents | OR: 0.020; 95%CI: 0.003, 0.037 | Negative[39] |
| Green space and urbanization | Built environment | COPD | Cross-sectional | Logistic regression | Urbanization was associated with a higher incidence of COPD, while residential green space was associated with a lower incidence of COPD | OR: 0.89; 95%CI: 0.84-0.93 | Urbanization: Positive, Residential green space: Negative[40] |
| Social economy and education | Social integration | CAD | Prospective cohort | Cox proportional hazards models | Social integration was found to contribute to a lower risk of CVD | HR: 0.67; 95%CI: 0.53, 0.86 | Negative[41] |
| Social economy and education | Social support | Dementia | Meta-analysis | Meta | A low social participation index was related to the risk of dementia, while a high social participation index had a moderately protective effect | RR: 1.59; 95%CI: 1.31, 1.96 | Negative[42] |
| Social economy and education | Educational level | Dementia | Cross-sectional | Logistic regression | Fewer years of education and being widowed, divorced, or living alone were risk factors for dementia. | OR: 1.55; 95%CI: 1.38, 1.73 | Negative[43] |
| Social economy and education | Household income | Dementia | Cross-sectional | Logistic regression | Compared with individuals with lower household income, those with a higher household income were less likely to receive a dementia diagnosis after referral | OR: 0.65; 95%CI: 0.55, 0.78 | Negative[44] |

**Abbreviations:** HR: hazard ratio; OR: odds ratio; RR: relative risk; CI: confidence interval; PM_2.5_: 2.5-µm particulate matter; PM_10_: 10-µm particulate matter; CO: carbon monoxide; SO_2_: sulfur dioxide; NO_2_: nitrogen dioxide; PFAS: per- and polyfluoroalkyl substances; PFOS: perfluorooctane sulfonate; PFOA: perfluorooctanoic acid; AD: Alzheimer’s disease; CAD: cardia-cerebrovascular diseases; CVD: cardiovascular disease; COPD: chronic obstructive pulmonary disease; PD: Parkinson’s disease; T2DM: type 2 diabetes mellitus

**References:**

1. Shin S, Burnett RT, Kwong JC, Hystad P, van Donkelaar A, Brook JR, Copes R, Tu K, Goldberg MS, Villeneuve PJ *et al*: **Effects of ambient air pollution on incident Parkinson's disease in Ontario, 2001 to 2013: a population-based cohort study**. *Int J Epidemiol* 2018, **47**(6):2038-2048.

2. Oudin A, Forsberg B, Adolfsson AN, Lind N, Modig L, Nordin M, Nordin S, Adolfsson R, Nilsson LG: **Traffic-Related Air Pollution and Dementia Incidence in Northern Sweden: A Longitudinal Study**. *Environ Health Perspect* 2016, **124**(3):306-312.

3. Chang KH, Chang MY, Muo CH, Wu TN, Chen CY, Kao CH: **Increased risk of dementia in patients exposed to nitrogen dioxide and carbon monoxide: a population-based retrospective cohort study**. *PLoS One* 2014, **9**(8):e103078.

4. Han C, Oh J, Lim YH, Kim S, Hong YC: **Long-term exposure to fine particulate matter and development of chronic obstructive pulmonary disease in the elderly**. *Environ Int* 2020, **143**:105895.

5. Li J, Sun S, Tang R, Qiu H, Huang Q, Mason TG, Tian L: **Major air pollutants and risk of COPD exacerbations: a systematic review and meta-analysis**. *Int J Chron Obstruct Pulmon Dis* 2016, **11**:3079-3091.

6. Shah AS, Lee KK, McAllister DA, Hunter A, Nair H, Whiteley W, Langrish JP, Newby DE, Mills NL: **Short term exposure to air pollution and stroke: systematic review and meta-analysis**. *BMJ* 2015, **350**:h1295.

7. Coogan PF, White LF, Jerrett M, Brook RD, Su JG, Seto E, Burnett R, Palmer JR, Rosenberg L: **Air pollution and incidence of hypertension and diabetes mellitus in black women living in Los Angeles**. *Circulation* 2012, **125**(6):767-772.

8. Andersen ZJ, Raaschou-Nielsen O, Ketzel M, Jensen SS, Hvidberg M, Loft S, Tjonneland A, Overvad K, Sorensen M: **Diabetes incidence and long-term exposure to air pollution: a cohort study**. *Diabetes Care* 2012, **35**(1):92-98.

9. Hansen AB, Ravnskjaer L, Loft S, Andersen KK, Brauner EV, Baastrup R, Yao C, Ketzel M, Becker T, Brandt J *et al*: **Long-term exposure to fine particulate matter and incidence of diabetes in the Danish Nurse Cohort**. *Environ Int* 2016, **91**:243-250.

10. Chen H, Burnett RT, Kwong JC, Villeneuve PJ, Goldberg MS, Brook RD, van Donkelaar A, Jerrett M, Martin RV, Brook JR *et al*: **Risk of incident diabetes in relation to long-term exposure to fine particulate matter in Ontario, Canada**. *Environ Health Perspect* 2013, **121**(7):804-810.

11. Yan D, Zhang Y, Liu L, Shi N, Yan H: **Pesticide exposure and risk of Parkinson's disease: Dose-response meta-analysis of observational studies**. *Regul Toxicol Pharmacol* 2018, **96**:57-63.

12. Baldi I, Lebailly P, Mohammed-Brahim B, Letenneur L, Dartigues JF, Brochard P: **Neurodegenerative diseases and exposure to pesticides in the elderly**. *Am J Epidemiol* 2003, **157**(5):409-414.

13. MP M, TM S, DP S, MC A, F K: **Incident Diabetes and Pesticide Exposure among Licensed Pesticide Applicators: Agricultural Health Study, 1993-2003**. *American Journal of Epidemiology* 2008(No.10):1235-1246.

14. Turyk M, Anderson H, Knobeloch L, Imm P, Persky V: **Organochlorine exposure and incidence of diabetes in a cohort of Great Lakes sport fish consumers**. *Environ Health Perspect* 2009, **117**(7):1076-1082.

15. Lee DH, Lind PM, Jacobs DR, Jr., Salihovic S, van Bavel B, Lind L: **Polychlorinated biphenyls and organochlorine pesticides in plasma predict development of type 2 diabetes in the elderly: the prospective investigation of the vasculature in Uppsala Seniors (PIVUS) study**. *Diabetes Care* 2011, **34**(8):1778-1784.

16. Coon S, Stark A, Peterson E, Gloi A, Kortsha G, Pounds J, Chettle D, Gorell J: **Whole-body lifetime occupational lead exposure and risk of Parkinson's disease**. *Environ Health Perspect* 2006, **114**(12):1872-1876.

17. Weisskopf MG, Weuve J, Nie H, Saint-Hilaire MH, Sudarsky L, Simon DK, Hersh B, Schwartz J, Wright RO, Hu H: **Association of cumulative lead exposure with Parkinson's disease**. *Environ Health Perspect* 2010, **118**(11):1609-1613.

18. Rondeau V, Jacqmin-Gadda H, Commenges D, Helmer C, Dartigues JF: **Aluminum and silica in drinking water and the risk of Alzheimer's disease or cognitive decline: findings from 15-year follow-up of the PAQUID cohort**. *Am J Epidemiol* 2009, **169**(4):489-496.

19. Sun Q, Zong G, Valvi D, Nielsen F, Coull B, Grandjean P: **Plasma Concentrations of Perfluoroalkyl Substances and Risk of Type 2 Diabetes: A Prospective Investigation among U.S. Women**. *Environ Health Perspect* 2018, **126**(3):037001.

20. Ongono JS, Dow C, Gambaretti J, Severi G, Boutron-Ruault MC, Bonnet F, Fagherazzi G, Mancini FR: **Dietary exposure to brominated flame retardants and risk of type 2 diabetes in the French E3N cohort**. *Environ Int* 2019, **123**:54-60.

21. Qiu C, Fratiglioni L, Karp A, Winblad B, Bellander T: **Occupational exposure to electromagnetic fields and risk of Alzheimer's disease**. *Epidemiology* 2004, **15**(6):687-694.

22. Linares C, Culqui D, Carmona R, Ortiz C, Diaz J: **Short-term association between environmental factors and hospital admissions due to dementia in Madrid**. *Environ Res* 2017, **152**:214-220.

23. Roswall N, Pyko A, Ogren M, Oudin A, Rosengren A, Lager A, Poulsen AH, Eriksson C, Segersson D, Rizzuto D *et al*: **Long-Term Exposure to Transportation Noise and Risk of Incident Stroke: A Pooled Study of Nine Scandinavian Cohorts**. *Environ Health Perspect* 2021, **129**(10):107002.

24. Gallo V, Vineis P, Cancellieri M, Chiodini P, Barker RA, Brayne C, Pearce N, Vermeulen R, Panico S, Bueno-de-Mesquita B *et al*: **Exploring causality of the association between smoking and Parkinson's disease**. *Int J Epidemiol* 2019, **48**(3):912-925.

25. Dawes P, Cruickshanks KJ, Moore DR, Edmondson-Jones M, McCormack A, Fortnum H, Munro KJ: **Cigarette smoking, passive smoking, alcohol consumption, and hearing loss**. *J Assoc Res Otolaryngol* 2014, **15**(4):663-674.

26. Klinedinst BS, Le ST, Larsen B, Pappas C, Hoth NJ, Pollpeter A, Wang Q, Wang Y, Yu S, Wang L *et al*: **Genetic Factors of Alzheimer's Disease Modulate How Diet is Associated with Long-Term Cognitive Trajectories: A UK Biobank Study**. *J Alzheimers Dis* 2020, **78**(3):1245-1257.

27. Patel CJ, Bhattacharya J, Butte AJ: **An Environment-Wide Association Study (EWAS) on type 2 diabetes mellitus**. *PLoS One* 2010, **5**(5):e10746.

28. Foubert-Samier A, Helmer C, Perez F, Le Goff M, Auriacombe S, Elbaz A, Dartigues JF, Tison F: **Past exposure to neuroleptic drugs and risk of Parkinson disease in an elderly cohort**. *Neurology* 2012, **79**(15):1615-1621.

29. Goldman SM, Quinlan PJ, Ross GW, Marras C, Meng C, Bhudhikanok GS, Comyns K, Korell M, Chade AR, Kasten M *et al*: **Solvent exposures and Parkinson disease risk in twins**. *Ann Neurol* 2012, **71**(6):776-784.

30. Gray SL, Anderson ML, Dublin S, Hanlon JT, Hubbard R, Walker R, Yu O, Crane PK, Larson EB: **Cumulative use of strong anticholinergics and incident dementia: a prospective cohort study**. *JAMA Intern Med* 2015, **175**(3):401-407.

31. Scheperjans F, Aho V, Pereira PA, Koskinen K, Paulin L, Pekkonen E, Haapaniemi E, Kaakkola S, Eerola-Rautio J, Pohja M *et al*: **Gut microbiota are related to Parkinson's disease and clinical phenotype**. *Mov Disord* 2015, **30**(3):350-358.

32. Vascellari S, Palmas V, Melis M, Pisanu S, Cusano R, Uva P, Perra D, Madau V, Sarchioto M, Oppo V *et al*: **Gut Microbiota and Metabolome Alterations Associated with Parkinson's Disease**. *mSystems* 2020, **5**(5).

33. Vogt NM, Kerby RL, Dill-McFarland KA, Harding SJ, Merluzzi AP, Johnson SC, Carlsson CM, Asthana S, Zetterberg H, Blennow K *et al*: **Gut microbiome alterations in Alzheimer's disease**. *Sci Rep* 2017, **7**(1):13537.

34. Walker KA, Gottesman RF, Wu A, Knopman DS, Gross AL, Mosley TH, Jr., Selvin E, Windham BG: **Systemic inflammation during midlife and cognitive change over 20 years: The ARIC Study**. *Neurology* 2019, **92**(11):e1256-e1267.

35. Li J, Lee DH, Hu J, Tabung FK, Li Y, Bhupathiraju SN, Rimm EB, Rexrode KM, Manson JE, Willett WC *et al*: **Dietary Inflammatory Potential and Risk of Cardiovascular Disease Among Men and Women in the U.S**. *J Am Coll Cardiol* 2020, **76**(19):2181-2193.

36. Savolainen-Peltonen H, Rahkola-Soisalo P, Hoti F, Vattulainen P, Gissler M, Ylikorkala O, Mikkola TS: **Use of postmenopausal hormone therapy and risk of Alzheimer's disease in Finland: nationwide case-control study**. *BMJ* 2019, **364**:l665.

37. Wu M, Li M, Yuan J, Liang S, Chen Z, Ye M, Ryan PM, Clark C, Tan SC, Rahmani J *et al*: **Postmenopausal hormone therapy and Alzheimer's disease, dementia, and Parkinson's disease: A systematic review and time-response meta-analysis**. *Pharmacol Res* 2020, **155**:104693.

38. Wu YT, Prina AM, Jones AP, Barnes LE, Matthews FE, Brayne C, Medical Research Council Cognitive F, Ageing S: **Community environment, cognitive impairment and dementia in later life: results from the Cognitive Function and Ageing Study**. *Age Ageing* 2015, **44**(6):1005-1011.

39. de Keijzer C, Tonne C, Basagana X, Valentin A, Singh-Manoux A, Alonso J, Anto JM, Nieuwenhuijsen MJ, Sunyer J, Dadvand P: **Residential Surrounding Greenness and Cognitive Decline: A 10-Year Follow-up of the Whitehall II Cohort**. *Environ Health Perspect* 2018, **126**(7):077003.

40. Sarkar C, Zhang B, Ni M, Kumari S, Bauermeister S, Gallacher J, Webster C: **Environmental correlates of chronic obstructive pulmonary disease in 96 779 participants from the UK Biobank: a cross-sectional, observational study**. *Lancet Planet Health* 2019, **3**(11):e478-e490.

41. Tan J, Wang Y: **Social Integration, Social Support, and All-Cause, Cardiovascular Disease and Cause-Specific Mortality: A Prospective Cohort Study**. *Int J Environ Res Public Health* 2019, **16**(9).

42. Penninkilampi R, Casey AN, Singh MF, Brodaty H: **The Association between Social Engagement, Loneliness, and Risk of Dementia: A Systematic Review and Meta-Analysis**. *J Alzheimers Dis* 2018, **66**(4):1619-1633.

43. Jia L, Du Y, Chu L, Zhang Z, Li F, Lyu D, Li Y, Li Y, Zhu M, Jiao H *et al*: **Prevalence, risk factors, and management of dementia and mild cognitive impairment in adults aged 60 years or older in China: a cross-sectional study**. *Lancet Public Health* 2020, **5**(12):e661-e671.

44. Petersen JD, Wehberg S, Packness A, Svensson NH, Hyldig N, Raunsgaard S, Andersen MK, Ryg J, Mercer SW, Sondergaard J *et al*: **Association of Socioeconomic Status With Dementia Diagnosis Among Older Adults in Denmark**. *JAMA Netw Open* 2021, **4**(5):e2110432.
